# On Entropy, Information, and Conservation of Information

**DOI:** 10.3390/e23060779

**Published:** 2021-06-19

**Authors:** Yunus A. Çengel

**Affiliations:** Department of Mechanical Engineering, University of Nevada, Reno, NV 89557, USA; yunus.cengel@yahoo.com

**Keywords:** entropy, information, conservation of information, creation of information, destruction of information, Boltzmann relation

## Abstract

The term *entropy* is used in different meanings in different contexts, sometimes in contradictory ways, resulting in misunderstandings and confusion. The root cause of the problem is the close resemblance of the defining mathematical expressions of *entropy* in statistical thermodynamics and *information* in the communications field, also called *entropy*, differing only by a constant factor with the unit ‘J/K’ in thermodynamics and ‘bits’ in the information theory. The thermodynamic property entropy is closely associated with the physical quantities of thermal energy and temperature, while the entropy used in the communications field is a mathematical abstraction based on probabilities of messages. The terms *information* and *entropy* are often used interchangeably in several branches of sciences. This practice gives rise to the phrase *conservation of entropy* in the sense of *conservation of information*, which is in contradiction to the fundamental *increase of entropy principle* in thermodynamics as an expression of the second law. The aim of this paper is to clarify matters and eliminate confusion by putting things into their rightful places within their domains. The notion of *conservation of information* is also put into a proper perspective.

## 1. Introduction

One needs to be cautious when dealing with information since it is defined differently in different fields such as physical sciences, biological sciences, communications, philosophy, and daily life. Generalization of a particular meaning into other fields results in incompatibilities and causes confusion. Therefore, clear distinction with precise language and mathematics is needed to avoid misunderstandings.

**Figure 1:**
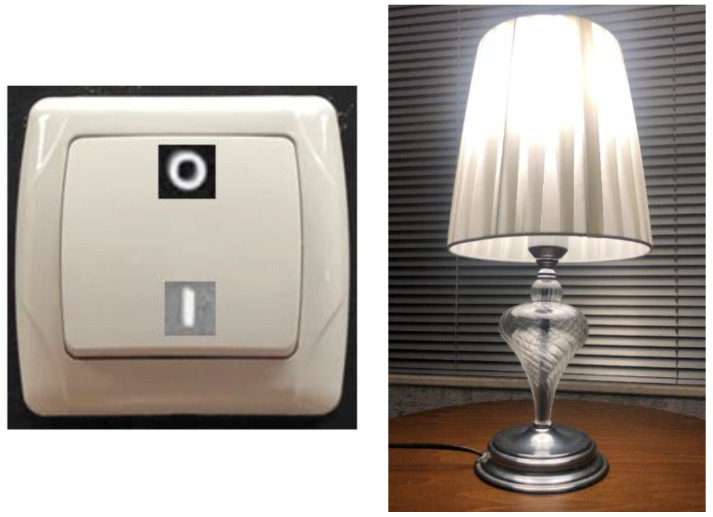
An electric lamp switch has two possible states only: on and off (or, 1 and 0). If the lamp is lit, we know that the switch is in the *on* position (Photos by Yunus Çengel).

The basic building block of modern electronics is the *transistor circuit*, which acts as a switch capable of staying in one of two states of on-or-off, or equivalently, closed-or-open, or 1-or-0 in the binary code, in response to an electrical signal input. A transistor functions in the same way as the light switch shown in Figure 1: the lamp turns on when the switch is moved to the *on* position and electric current is allowed to pass, and the lamp turns off when the switch is moved to the *off* position.

In electronics, a single switch or transistor constitutes the most elementary hardware. This basic case of one elementary component *n* = 1 corresponds to two sates of on and off, *M* = 2. In the area of communication via signal transmission, the information *I* associated with a single basic switch being on or off corresponds to a binary digit, and thus to a choice between two messages, and is taken as the unit of information called a *bit*. Therefore, 1 bit represents the amount of information that can be stored by a single switch with two stable states or positions of on/off, closed/open, or 1/0. The bit also represents the basic unit of entropy for a system that has only two states. 

If one switch corresponds to one bit of information, we intuitively feel that information should be additive, and three switches should correspond to three bits of information. However, in the case of three switches, there are 2^3^ = 8 possible choices or combinations of 111, 110, 101, 100, 001, 010, 011, and 000 for messages, the last choice corresponding to the case of all three switches being off. 

Therefore, as the number of switches increases linearly, the number of possible messages, which is equivalent to the amount of information that can be conveyed, increases exponentially. To keep things simple and manageable, it is desirable to define information in such a way that the amount of information is linearly related, and thus directly proportional, to the amount of hardware—the number of switches or transistors in this case. After all, the amount of hardware is a definitive indicator of the amount of information. This way, the amount of hardware becomes a measure of information, which is very practical if we are to build communication systems with various numbers of transistors or switches.

**Figure 2:**
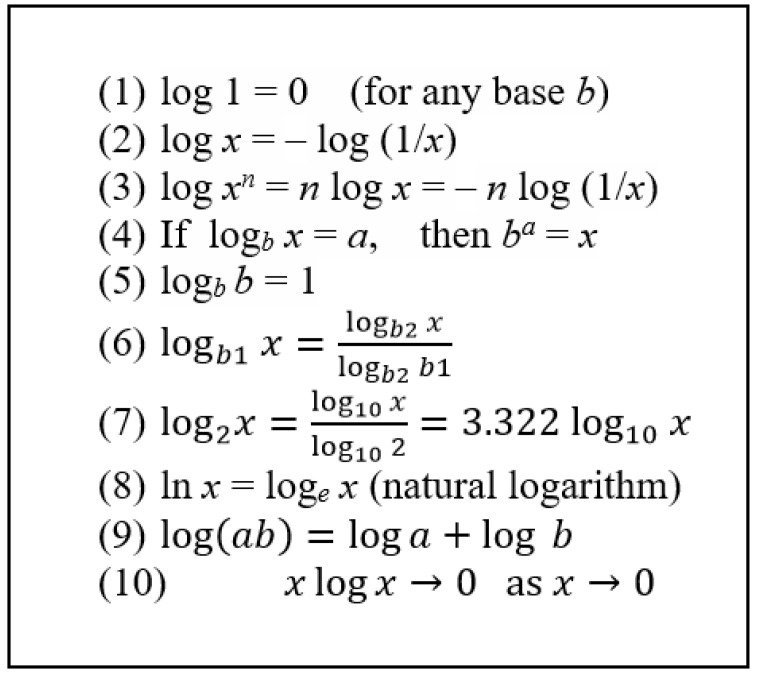
Some mathematical relations regarding logarithms (no specified base indicates any base).

A convenient and intuitive way of quantifying information is to relate it linearly to the number of switches so that information doubles when the number of switches is doubled. The simplest way of doing this is to equate one switch to one unit of information. Following the suggestion of Hartley [1], Shannon [2] and Weaver [3] have formulated this idea by defining information *I* associated with *n* switches as the logarithm to the base 2 of the number of possible choices or messages *N*:

*I* = log_2_*N* = log_2_ 2*^n^* = *n* log_2_ 2 = *n* (bits) (1)

since log_2_ 2 = 1, as shown in Figure 2. In the case of a single switch, *n* = 1 and thus information is *I* = 1 bit. Then, as desired, the information associated with *n* = 3 switches becomes

*I* = log_2_ *N* = log_2_ 8 = log_2_ 2^3^ = 3 log_2_ 2 = 3 bits (2)

The unit *bit* may appear obscure and arbitrary at first. However, this is also the case for many familiar units such as meter, gram, and second. Besides, it is common to use constant multiples of a unit in place of a unit itself, such as using kilogram instead of gram as a unit of mass and using *byte* (equivalent to eight bits) or even gigabytes (1 billion bytes) instead of *bit* as a unit of information and storage. Additionally, note from Figure 2 that log_2_ *x* = 3.322 log_10_ *x* and thus the logarithm of any base can be used in information calculations in practice, including a natural logarithm, by incorporating a suitable constant factor of multiplication. 

A more general and practical approach is to define information in terms of *probabilities p* of messages instead of the number of choices for messages, as presented next. Noting that the probability of a choice or message in this *equiprobable* case with three switches is *p* = 1/*N* = 1/8,

*I* = log_2_ *N* = log_2_ (1/*p*) = − log_2_ *p* = − log_2_ (1/8) = log_2_ 2^3^ = 3 bits (3)

since again, log_2_ 2 = 1. 

It is important to make the distinction that information as defined here is related to the *number of possible different messages* or *configurations* associated with a system, not *how much we know* about the system. Unlike the intuitive meaning in common usage, information in the communications field is used as a measure of the *choices* we have for a message configuration. Information associated with a message is closely related to the probability of that message. In fact, in a counterintuitive sense, the more we know about a system, the less remains to be known about that system, the less the uncertainty, the fewer the choices, and thus the smaller the information.

Information as used in the communication theory is of a statistical nature, and it represents probabilities. When we roll a fair dice with perfect symmetry, for example, the probability of landing 2 (or any other number between 1 and 6) is simply 1/6. The sum of all probabilities is 1, which is expressed as the conservation of information. Actually, what is conserved is the *space of possibilities* or the *search space*. If you win when an even number lands, your chance of winning when a dice is rolled is 1/2 or 50 percent. An apparent outcome of the conservation of information is that additional information increases the probability of success in achieving the objective—raising it from 1/6 to 3/6 in this case. 

## 2. Information Expressed as Probabilities of Messages

In sciences and engineering, it is usually more instructive to work with quantities that are normalized using a common base. In information science, this is done by using *probabilities* of the messages since probabilities are positive numbers between zero and one (0 ≤ *p*_i_ ≤ 1), and the sum of all probabilities associated with an event is one (Σ*p*_i_ = 1). Symmetry in configuration dictates equality in probabilities. 

In the simple case of *equal probabilities* of a total of *N* messages (or an event horizon of *N*), the probability *p* for the occurrence of a particular message is simply *p* = 1/*N*, as stated above. In the case of a single switch, for example, *N* = 2 and the probability of the switch being ‘on’ (or ‘1’) or ‘off’ (or ‘0’) is *p* = ½, just like the probability of landing heads or tails when a fair coin with perfect symmetry is flipped is *p* = ½. Noting that *p* = 1/*N* = ½, the information associated with an equiprobable two-message or two-state case is

*I* = log_2_ *N* = log_2_ (1/*p)* = − log_2_ *p* = − log_2_ (1/2) = log_2_ 2 = 1 bit (4)

The negative sign in this expression should not cause any concern since probabilities associated with a system or an event are always positive numbers in the range of 0 ≤ *p* ≤ 1, and logarithms of positive numbers less that unity are always negative. The two negative signs cancel each other out and ensure that information is a positive quantity. Here, a person is equally free to choose one of the two alternative messages. 

In the case of a two-state system such as the single switch or the coin discussed here, a convenient way of rewriting the information relation *I* = −log_2_ *p* is to express it as the sum of the information associated with each state *weighed* by the probability of that state. Noting that *p*_1_ = *p*_2_ = ½ in this case, the information associated with this equiprobable two-state system can be expressed as

*I* = − (*p*_1_log *p*_1_ + *p*_2_log *p*_2_) (5)

and thus

*I* = − (½ log ½ + ½ log ½) = − (½ + ½ )log ½ = − (1)(−1) = 1 bit (6)

since log ½ = −1. The importance of this relation is that it turns out to be valid even when the probabilities *p*_1_ and *p*_2_ are not equal to each other. For example, when a switch is stuck at the ‘on’ (or ‘closed’) position and cannot be turned off, we have *p*_1_ = 1, *p*_2_ = 0 and thus

*I* = − (*p*_1_log *p*_1_ + *p*_2_log *p*_2_) = − (1 × log 1 + 0 × log 0) = 0 (7)

which indicates *full knowledge* of the state of a switch and thus zero information or uncertainty (entropy) for that system. That is, *zero information* in the information theory corresponds to *full knowledge* and thus *zero ignorance*. 

We obtain the same result when the switch is held at the ‘off’ (or ‘open’) position and thus *p*_1_ = 0 and *p*_2_ = 1. Therefore, when there is no freedom of choice, as in the case of a switch always being ‘on’ and thus the probability of ‘on’ is 1 (certainty) and the probability of ‘off’ is 0 (impossibility), there is no information or uncertainty since there is no choice, and thus information is zero. This is similar to the entropy of a perfect crystalline substance being zero at absolute zero temperature (the third law of thermodynamics) since all atomic motions supposedly come to a halt at that moment and thus there is only one possible microstate for the constituents (no uncertainty). 

For a general two-state system of variable probabilities, the probabilities of the two states can be expressed as *p*_1_ = *p* and *p*_2_ = 1 − *p*_1_ = 1 − *p* and thus, from Equation (5),

*I* = − (*p*_1_log *p*_1_ + *p*_2_log *p*_2_) = − [*p* log *p* + (1 − *p*) log (1 − p)] (8)

**Figure 3:**
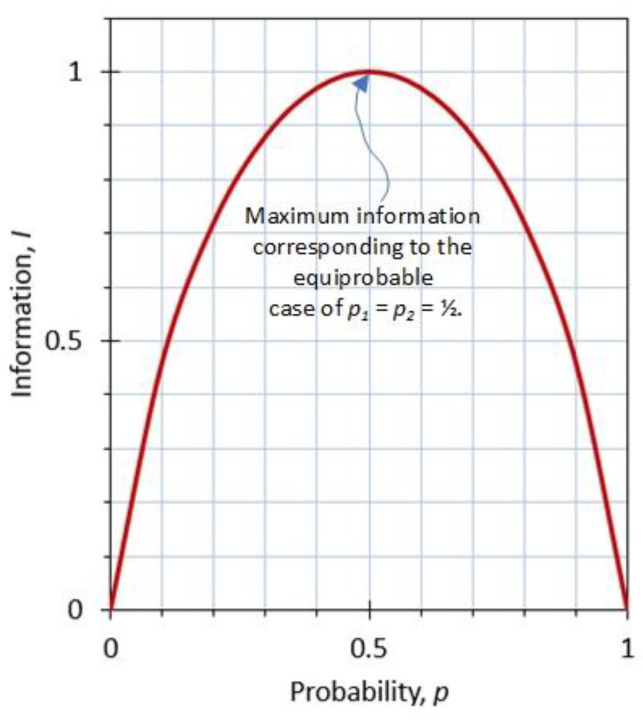
The variation of information (or entropy) for a two-state and thus a two-probability system as *p*_1_ = *p* varies between 0 and 1 (and *p*_2_ = 1 − *p*).

Taking the derivative of *I* with respect to the variable *p*, setting it equal to zero, and solving for *p* gives *p* = 1/2, which corresponds to a point of extremum at which the value of *I* is a maximum. This indicates that for a two-state system of variable probabilities, information *I* = 0 when *p*_1_ = 1 and *p*_2_ = 0 increases as *p*_1_ decreases and *p*_2_ increases, reaches a maximum of *I* = 1 when *p*_1_ = *p*_2_ = 1/2, decreases as *p*_1_ continues to decrease and *p*_2_ continues to increase, and finally *I* = 0 when *p*_1_ = 0 and *p*_2_ = 1, as shown in Figure 3. 

Note that information *I* reaches its maximum value when the probabilities of all states are equal (*p*_1_ = *p*_2_ = ½). This corresponds to the case of maximum information, maximum uncertainty, and maximum entropy (and minimum knowledge). That is, when there are many choices, information *I* is the largest when the probabilities of choices are nearly uniformly distributed, making every choice almost equally viable. On the other hand, information *I* is smallest when one of the choices has a probability of nearly one, rendering other choices nil and thus leaving little freedom of choice and little uncertainty. 

The general form of the information relation expressed above for a basic two-state system in terms of probabilities is not limited to a system with just two messages or configurations. For a communication system that involves a set of *N* independent complete messages (or a physical system with *N* possible configurations) indexed by *i*, the information expression above can be generalized as

(9) $$I=-(p_{1}\;\log\;p_{1}+p_{2}\;\log\;p_{2}+\ldots +{p_{N}}_{\;}\log\;p_{N})=-\sum_{i=1}^{N}p_{i}\log p_{i}$$

or simply,

(10) $$I=-\sum^{\;}p_{i}\log p_{i}$$

where *p_i_* is the probability of the message or configuration *i*. Here, we dropped the symbol for base 2 for simplicity. In the case of equal probabilities for all *N* messages of configurations, we have *p_i_* = 1/*N* = constant, and the general relation in Equation (10) reduces to

*I* = − Σ*p_i_* log *p_i_* = – Σ*p_i_* log (1/*N*) = (Σ*p_i_*) log *N* = (1) log *N* = log *N* (11)

since the sum of all probabilities associated with an event is unity and thus Σ*p_i_* = 1 (conservation of information). 

Note that we could define information (also called entropy) from the start as *I* = − Σ*p_i_* log *p_i_* if we wanted to and obtain the relation *I* = log *N* as a special case corresponding to the case of all probabilities being equal to each other and thus *p_i_* = constant. However, the step-by-step approach presented above is more intuitive and pedagogically more sound. 

Note that *p_i_* log *p_i_* → 0 as *p_i_* → 0, as given in Figure 2. Therefore, the states or messages with very small probability contribute very little to information (or entropy), and the contribution of states with zero probability (unoccupied states) is zero. Finally, in the case of a continuum where probabilities vary as a function *f*(*p*) instead of being specified as a set of discrete values *p*_1_, *p*_2_, … *p_N_*, the *summation* in the general information relation above can be replaced by *integration* as

*I* = – ∫*p* log *p dp* (12)

The quantity $\sum^{\;}p_{i}\log p_{i}$ is unitless since both the probabilities and logarithms are unitless. The magnitude of the unit of a newly defined quantity can be selected arbitrarily, like selecting a certain length and calling it ‘1 meter’. Information can also be expressed to reflect a choice for the unit of measure by incorporating a constant positive multiplication factor *k* as

(13) $$I=-k\sum^{\;}p_{i}\log p_{i}$$

Here, the unit of the constant *k* becomes the unit of *I*, and *p*_1_, *p*_2_, … *p_N_* are the set of probabilities associated with a state, a configuration, or an event. The unitless quantities log *N* and *p_i_*log *p_i_* merely indicate magnitudes. 

In statistical thermodynamics, the constant *k* in Equation (13) is the Boltzmann constant with the unit of J/K, as discussed below, and *I* in that case represents the thermodynamic entropy, *S*. In information theory, *k* is simply taken to be *k* = 1 bit, and *I* represents informational entropy or just information, message, choice, or uncertainty:

(14) $$I=S=-\sum^{\;}p_{i}\log p_{i}$$

Information *I* is a positive quantity except when all *p_i_* other than one of them are zero, and the nonzero *p_i_* necessarily has a value of 1 since Σ*p_i_* = 1. This represents the case of *I* = 0, which happens when the outcome of an event is certain. 

In daily life, *information* and *knowledge* are often used interchangeably as synonyms. However, in information science, they are used as *antonyms*: zero information means complete knowledge and thus zero ignorance while maximum information corresponds to minimum knowledge and thus maximum ignorance.

A *transmitter* accepts the messages or symbols of information, such as written or spoken words, and turns them into signals via the process of coding. Thus, a transmitter encodes messages while a *receiver* decodes them. The number of bits actually transmitted per second constitutes the *transmission rate* of information. The number of bits that can be transmitted through a communication channel per second constitutes the *capacity* of that channel.

The introduction of *noise* during transmission distorts the message and increases uncertainty, which in turn increases information, making it more difficult to distill out and process the useful information in the received signal. A high value of uncertainty and thus information at the sending end is desirable since it means more freedom of choice. However, the increase of information and thus uncertainty at the receiving end due to the influence of noise in the received signal is undesirable. 

When there are no interventions from noise or other causes, which would be the case during transmission over a noiseless channel, there is no *information (or entropy) generation* during transmission. The sent and received signals and the corresponding symbols of information in this idealized case would be identical, and information (or entropy) would be conserved. When noise and other interventions are introduced, the probabilities of the symbols received will change. Under good transmission conditions, the changes in probabilities of received symbols relative to the sent symbols will be small. Once the probabilities are known, the entropies associated with messages can be calculated. The difference between the information (or entropy) of received signals and the uncertainty in the received signal due to noise is the useful information transmitted.

### A Novel Three-Lamp Information System 

**Figure 4:**
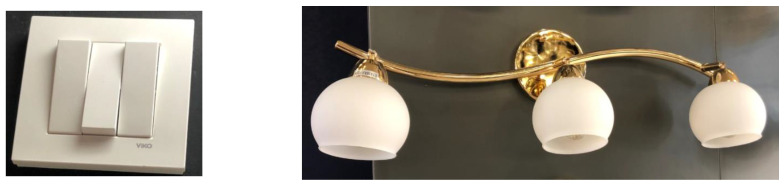
A system of three switches controlling three lamps (Photos by Yunus Çengel).

Consider a system of *n* = 3 light switches, as shown in Figure 4, controlling three separate lamps. Each of the switches can be on or off, or equivalently 1 or 0, and thus each switch has *M* = 2 possible states. Also, the system of three switches has 3 degrees of freedom since each switch is free to assume one of the two different positions or states. 

The possible on/off combinations of these three switches that constitute the *configuration space* of the system are 111, 110, 101, 100, 001, 010, 011, and 000—the last one corresponding to the case of all three switches and thus all the lamps being off. Therefore, the total number of possible configurations of the three switches or lamps is

*N* = *M ^n^* = 2^3^ = 8 (15)

That is, we have eight possible choices for the on/off combinations of the three lamps, like keeping the first lamp off while turning the second and third lamps on (011). 

We certainly can use the sequence of the on/off combinations of the three lamps in our room for messaging to communicate with the neighborhood. For example, the first lamp off and the other two lamps on (011) may mean ‘studying’ while all lamps off (000) may signal sleeping. Of course, the neighbors should be aware of the meaning a particular sequence of lighting corresponds to. Therefore, using a system of three lamps, we can have a communication system with eight possible choices for messages, or equivalently, eight possible pieces of information. If we double the number of lamps from 3 to 6, the number of possible choices for messages will increase from 8 to *N* = 2*^n^* = 2^6^ = 64. 

The hardware of our primitive information system for communication is comprised of lamps, and as the number of lamps increases linearly, the number of possible messages, which represents the amount of apparent information that can be conveyed, increases exponentially. Information is defined above in such a way that the amount of information is linearly related and thus directly proportional to the amount of hardware (the number of lamps or switches in this case). Because of linearity, *n* switches can store *n* bits of information, as the relation *I* = *n* indicates. Note that the number of bits of a system also represents the information of the system (or equivalently, its entropy). Then, the nominal information or entropy of this three-switch system is *I* = *S* = *n* = 3 bits. 

As stated above, information associated with a message is intimately related to the probability of that message. The more we know about a system, the less remains to be known about that system, the less the uncertainty, the fewer the choices, and thus the smaller the information. If the first switch is always to be kept on, for example, we would have *n* = 2 instead of *n* = 3 in the equation above, and thus less information and fewer choices.

The three-switch system analyzed here with *M* = 2 represents the *least restrictive case* since the switches are free to be on or off, and corresponds to the maximum information (and maximum entropy) and zero knowledge about the states of switches. Now consider the *most restrictive case* with all three switches fixed at the ‘on’ state and thus *M* = 1. In this case, the state of this three-switch system is fully known, and there is only one state or configuration, which is 111. Then, *N* = 1 and

*I* = log_2_*N* = log_2_ 1 = 0 = *S* (16)

That is, *full knowledge* of the state of a system corresponds to *zero information* and *zero entropy* for that system, and a probability of 1 for the configuration 111. So, in general, the more is known about the system, the smaller the entropy and information. 

In a hybrid case of two of the switches being kept on and one switch off but not knowing which one, there are three possible states or configurations, which are 110, 101, and 011. Then, *N* = 3 and

*I* = log_2_*N* = log_2_ 3 = 1.585 bits = *S* (17)

Therefore, information or entropy decreases from 3 bits to 1.585 bits when any two of the switches are kept on while the remaining one is off. It seems that as the definiteness or determinacy of a system increases, information (or entropy) decreases. 

It is apparent from the simple cases discussed above that information in the communication field is a measure of freedom of choice. In the absence of noise, information is conserved since the number of choices during transmission is fixed.

The probabilistic nature of information in the communication field and the quest for a high probability of accuracy in the transmission of information also has intriguing ontological ramifications. As William Dembski [4] puts it, “*Any scene, indeed any input to our senses, reaches our consciousness only by aspects becoming salient, and this happens because certain patterns in our background knowledge are matched to the exclusion of others. In an extension of George Berkeley’s ‘to be is to be perceived,’ conservation of information suggests that ‘to be perceived is to be an object of search.’ By transitivity of reasoning, it would then follow that to be is to be an object of search. And since search is always search for an object, search and the object of search become, in this way of thinking, mutually ontologizing, giving existence to each other. Conservation of information then adds to this by saying that search can itself be an object of search*”.

## 3. Entropy in Statistical Thermodynamics

Entropy can be viewed as a measure of molecular disorder, or molecular randomness. As a system becomes more disordered, the positions of the molecules become less predictable and the entropy increases. Thus, it is not surprising that the entropy of a substance is lowest in the solid phase and highest in the gas phase. In the solid phase, the molecules of a substance continually oscillate about their equilibrium positions, but they cannot move relative to each other, and their position at any instant can be predicted with good certainty. In the gas phase, however, the molecules move about at random, collide with each other, and change direction, making it extremely difficult to predict accurately the microscopic state of a system at any instant. Associated with this molecular chaos is a high value of entropy.

**Figure 5:**
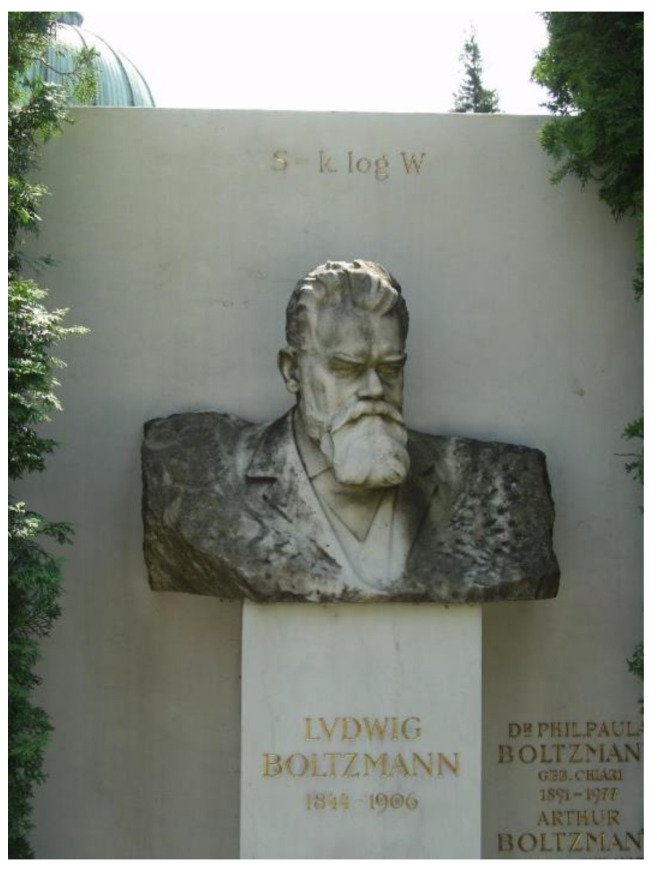
Boltzmann’s tombstone engraved with his famous formula *S* = *k* log *W* (In the case of evenly distributed probabilities over all the occupied states of a system, entropy is a constant multiple of the logarithm of the number of the occupied states, *W*). (Credit: Tom Schneider, https://alum.mit.edu/www/toms/images/boltzmann (accessed on 1 June 2021); public domain.).

When viewed microscopically from a statistical thermodynamics point of view, an isolated system that appears to be at a state of equilibrium actually exhibits a high level of activity because of the continual motion of the molecules. To each state of macroscopic equilibrium, there corresponds a large number of molecular microscopic states or molecular configurations. Boltzmann first hypothesized that the entropy of a system at a specified macrostate is related to the total number of possible relevant microstates of that system, *W* (from *Wahrscheinlichkeit*, the German word for ‘probability’). This thought was formulated later by Planck using a constant *k* with the entropy unit of J/K named after Ludwig Boltzmann (1844–1906) and inscribed on Boltzmann’s tombstone (Figure 5) as

*S* = *k* ln *W* (18)

which resembles Equation (1) for information and is known as the *Boltzmann relation*. Here, *k* = 1.380650 × 10^−23^ J/K is the *Boltzmann’s constant*. The thermal motion randomness or disorder, as related to entropy, was later generalized by Josiah Willard Gibbs (1839–1903) as a measure of the sum of all microstates’ uncertainties, i.e., probabilities, as [5]

*S* = − *k* Σ*p_i_* log *p_i_* (19)

which is identical to Equation (13) for information, differing only in the value of the constant *k*. The *Gibbs’ formulation* in Equation (19) is more general since it allows for a non-uniform prob ability, *p_i_*, of microstates. With an increase of particle momenta or thermal disorder and volume occupied, more information is required for the characterization of the system, relative to more ordered systems. 

From a microscopic point of view, the entropy of a system increases when ever the thermal randomness or disorder (i.e., the number of possible relevant molecular microstates corresponding to a given bulk macrostate) of a system increases. Thus, entropy can be viewed as a measure of thermal randomness or molecular disorder, which increases any time an isolated system undergoes a process. 

The molecules of a substance in solid phase continually oscillate, creating an uncertainty about their position. These oscil lations, however, fade as the temperature is decreased, and the molecules supposedly become motionless at absolute zero. This represents a state of ultimate molecular order (and minimum energy). Therefore, the entropy of a pure crystalline substance at absolute zero temperature is zero since there is no uncertainty about the state of the molecules at that instant. This statement is known as the *third law of thermodynamics*.

The third law of thermodynamics provides an absolute reference point for the deter mination of entropy. The entropy determined relative to this point is called *absolute entropy*, and it is extremely useful in the thermodynamic analysis of chemical reactions. Notice that the entropy of a substance that is not pure crystalline (such as a solid solution) is not zero at absolute zero temperature. This is because more than one molecular configuration exists for such substances, which introduces some uncertainty about the microscopic state of the substance [6].

## 4. Information and Entropy

Warren Weaver, one of the founders of the information theory, stated in his seminal 1949 paper that “Information is, we must steadily remember, a measure of one’s freedom of choice in selecting a message. The greater this freedom of choice, and hence the greater the information, the greater is the uncertainty that the message actually selected is some particular one. Thus, greater freedom of choice, greater uncertainty, greater information go hand in hand”. 

The resemblance of information in this context and the thermodynamic property *entropy* is apparent. After all, *entropy* is a measure of uncertainty, and the larger the uncertainty, the larger is the entropy. Further, information is expressed using an entropy-like expression. However, although information within the context of the communication field is usually conserved, the entropy of energy systems is not except for the idealized cases of reversible processes (the second law of thermodynamics).

Entropy *S* was defined above for a uniform system by the Boltzmann relation (Equation (18)) as

*S = k* ln *W*

Here, *k* is the Boltzmann’s constant and *W* is the total number of possible microstates of the system corresponding to a bulk macrostate at a given temperature and pressure, which is equivalent to the uniform probability of all possible *W* microstates. For a nonuniform system comprised of *n* uniform subsystems indexed by *i*, the entropy was given by the more general Gibbs formulation (Equation (19)) expressed as

*S = – k* Σ*p_i_* log *p_i_*

For a continuum of volume *V*, it is expressed in the integral form as *S =* − *k*∫*p* log *p* d*p*. Here, *p_i_* is the probability of a microstate in subsystem *i*, which is the inverse of the total number of equiprobably microstates of subsystem *i*. In the case of an equiprobable, uniform probability of all *W* microstates, the Gibbs formulation of entropy reduces to the Boltzmann relation,

*S =* − *k* Σ*p_i_* log *p_i_* = − *k* (1) log (1/*W*) = *k* log *W* (20)

since *p_i_* = 1/*W* = constant ≪ 1 and Σ*p_i_* = 1. This was also the case for information.

Entropy is a physical quantity and has the unit J/K, where J is the energy unit *joule* and K is the temperature unit *kelvin*. Information, on the other hand, is a mathematical quantity and has no physical units—the unit ‘bit’ is an abstract mathematical quantity. 

In statistical thermodynamics, *entropy S* is proportional to the logarithm of the number of possible configurations *N* of the *n* components of a system. For a *M* = 2-state component, entropy, like information, is expressed as

*S* = *k* log *N* = *k* log *M ^n^* = *k* log 2*^n^* = *n k* log 2 (21)

This relation is identical in form to the expression for information given in Equation (1) when *N* is taken to be the number of messages and the constant of proportionality is taken to be *k* = 1. This explains why information and entropy are used interchangeably in the physical and information sciences, and why the unit of information *bit* is also used as the unit of entropy. Additionally, both entropy and information are proportional to the degrees of freedom or the number of switches *n* that comprise the system in this simple case.

Although information and entropy share the same equations, the fact remains that the units ‘bit’ and J/K in the constant *k* are very different, and thus information and entropy remain as completely different quantities. The differential equations of electric current flow and heat flow are also identical, differing only in a constant with different units. However, this is sufficient to view heat and electric charge as different quantities and never mix them, although the same computer code is used to solve both problems numerically for convenience. 

Information (or entropy) in physical sciences and in the communications field is proportional to the number of possible states or configurations *N* with non-zero probability. At a given time, the probability of any of the possible states of an equiprobable system is *p* = 1/*N*. These possible states may be reshuffled as time progresses. The larger the number of allowed states *N* is, the larger the information, the larger the uncertainty or the degrees of freedom to keep track of, and thus the larger what is not known. Therefore, ironically, information in physical and information sciences turns out to be a measure of ignorance, not a measure of knowledge, as explained before. The maximum value of information (or entropy) in this case is *I*_max_ = log *N*, which shows that the maximum information (or entropy) is representative of the number of states or configurations in a system. In the special case of no loss or gain of information and thus information or entropy is conserved, the occupied states are reshuffled while the total number of states remains constant.

We can eliminate the constant multiple ‘log 2’ in Equation (1) by using a logarithm to the base 2 rather than base 10:

*I* = log_2_*N* = log_2_ 2*^n^* = *n* log_2_ 2 = *n* = *S * (since log_2_ 2 = 1) (22)

For a system of *n* switches or components, the value of *n* log 2 represents the *maximum information I*_max_ as well as the *maximum entropy S*_max_. This corresponds to *maximum ignorance* and thus *minimum knowledge* (zero in this case) about the state of the system, and thus the most uncertain situation. This limiting case occurs when the probabilities of all possibilities are equal. Note that information in the communications field is the opposite of knowledge in common usage. Full knowledge (or certainty) of the state or configuration of the system corresponds to *zero information* (*I* = 0) or *zero entropy* (*S* = 0). This limiting case occurs when the probabilities of all possibilities are zero, except one that has a value of unity. This corresponds to certainty of the outcome of an event. For all other cases, *I* or *S* is a positive quantity.

Entropy is an *additive* quantity, just like energy. The entropy of a system at a given state is the sum of the entropies of the constituents of the system, just like the energy of the system is equal to the sum of the energies of the constituents of the system. The additive nature of entropy at a given state should not be confused with the conservation of entropy since the concept of conservation is associated with a change of state or process, not a particular state. 

When an isolated system undergoes an internal change (such as a hot rock in the system cooling down by transferring heat to the adjacent cooler constituents, for example), the total energy content of the system remains constant while the entropy increases as a result of the irreversibilities that occur during the process. This is why we have the *conservation of energy principle* and the *increase of entropy principle* in thermodynamics. Energy, entropy, and information are all additive. However, energy is always conserved during a process, information (or informational entropy) is conserved only during transmission over noiseless channels, and thermodynamic entropy is almost always generated during a process except for the rare cases of reversible processes such as superconductivity.

Due the close resemblance in their mathematical definitions (albeit without Boltzmann’s constant), the common practice of using information and entropy interchangeably has the potential to cause misunderstandings. To avoid confusion, it should be remembered that although the formulations of statistical thermodynamic entropy and informational entropy differ only by a constant factor, they represent different quantities. 

Information or entropy is related to the probability distribution associated with the internal structure or the configuration of the constituents of a system and represents the degree of incompleteness of our knowledge about the precise state of the system under consideration. In classical thermodynamics, we describe the macroscopic state of a system in equilibrium by measuring properties such as temperature, pressure, and volume, and calculate the rest of the properties such as entropy and enthalpy by using property relations. This certainly has proven to be very convenient, and it works well in practice. However, whether we realize or not, the measured values of macroscopic properties such as temperature and pressure are the manifestations of the statistical average of the behavior of a large number of molecules that constitute the system. In addition, the apparent behavior of the system is the outcome of the probabilistic description of the state of the system at the microscopic level in terms of positions and velocities of the constituents. 

In statistical thermodynamics, the aggregate properties of the system such as the temperature, pressure, and internal energy at a state are related to entropy, which is a measure of the statistical information in terms of probabilities over a large number of possible configurations of positions and velocities of the constituents at that state. Temperature and pressure are simply the *indicators* of the levels of the aggregate molecular activities, and there is a definite one-to-one correspondence between them. As such, we can determine the entropy of a gas in a closed system, for example, by simply measuring its temperature and pressure. The state postulate dictates that once two independent intensive properties for a simple compressible system are fixed, all other properties of the system must also be fixed and thus must assume definite values. The classical approach misses the details of activities at the constituent level but gives us all the information we need at the system level.

To have a good understanding of information and statistical thermodynamics, it is necessary to have a higher-level understanding of the fundamentals of statistics and probabilities. For example, using the ten digits of 0 through 9, the number of six-digit numbers we can possibly write is 10^6^ or one million. Likewise, the number of three-letter words we can write using the 26-letter English alphabet is 26^3^ = 17,576. Therefore, when three letters are drawn randomly, the probability of getting a meaningful sequence of letters such as ‘the’ is the same as the probability of ending up with a meaningless sequence such as ‘xqe’, which is p = 1/17,576 for each. When randomly withdrawing a three-letter word from an English book, however, the probability of getting the word ‘the’ is much higher than 1/17,575 while the probability of getting the word ‘xqe’ is practically zero. This is because only a limited number of three-letter sequences are meaningful in English, and the usage frequency is different for different words. This distinction provides the backdrop for efficiency and accuracy in the transmission and storage of information and shows the importance of dealing with a non-uniform distribution of probabilities.

## 5. Conservation of Information

The notion of *conservation of information* in physical sciences often causes confusion in this age of information when new information is being generated at an ever-increasing rate, doubling every few months. The near-synonymous use of knowledge and information in daily life and the intuitive sense that knowledge is limitless adds to the confusion associated with the notion of conservation of information. Obviously, there is a need to provide some clarity and set clear boundaries, and put things into a proper perspective. Once different definitions of information are put into their rightful places while preserving the distinct nature of each, much of the confusion should disappear.

Any person with a rudimentary knowledge of physics is familiar with the concept of conservation of physical entities, such as the conservation of energy, also known as the first law of thermodynamics. However, the notion of information being conserved, with the implication that there is a fixed amount of information in the universe, is hard to grasp since we are overwhelmed by the amount of new information as we understand it being created every day, and the continual acquisition of knowledge. Technological marvels such as satellites and smart phones as well as millions of patent applications every year are indicative of the new information generated. 

A fire at a crime scene often destroys crucial information that would lead to the prosecution of the suspects. Information related to many species that went extinct is also destroyed. Furthermore, in the communication field, information is viewed as synonymous with entropy, which is a non-conserved quantity per the second law of thermodynamics. In light of the colossal amounts of information being created and destroyed, it is fair to say that the concept of conservation of information is not general, and it must be confined to a particular form of information in a particular field.

In physical sciences, information is taken to be a fundamental ingredient of the universe, just like matter and energy. The notion of conservation of information is limited to the domain of physical sciences. Even in this limited scope it is not free of controversy, as the debates on Hawking’s black hole paradox indicate. Ironically, the conservation of physical information is based on the mathematical representation of information as entropy, which, in thermodynamics, is a non-conserved quantity.

However, in information theory, entropy is associated with the probability distribution, and thus we speak of the conservation of entropy: if there is no information lost or new information generated, then informational entropy is conserved. When there is no uncertainty and thus the state or configuration of a physical system is fully known, the informational entropy is zero. 

The state or configuration of particles in a system can be likened to the configuration of the playing cards in a deck. Reshuffling changes the occupied states and thus the configuration of the cards in the deck. However, the probabilities of the cards remain intact, and thus information is conserved. Note that uncertainty associated with information has nothing to do with Heisenberg’s uncertainty principle in quantum mechanics. 

Although the use of the phrase *conservation of information* is fairly recent [7], the basic idea can be traced to the French mathematician and astronomer Pierre Simon Laplace (1749–1827), who is a strict determinist: “*We may regard the present state of the universe as the effect of its past and the cause of its future. An intellect which at a certain moment would know all forces that set nature in motion, and all positions of all items of which nature is composed, if this intellect were also vast enough to submit these data to analysis, it would embrace in a single formula the movements of the greatest bodies of the universe and those of the tiniest atom; for such an intellect nothing would be uncertain and the future just like the past would be present before its eyes*” [8].

This deterministic framework sets the stage for the notion of the conservation of information, which should be viewed against this backdrop that the past and the future being determined precisely by the present state of the universe in strict compliance with the universal laws of physics and the associated push–pull effects of the forces of physics. In the case of conservation of information, the laws and forces of physics are augmented, sometimes even replaced, by information. Conservation of information implies that the information contained within the current moment is sufficient to determine every other moment—past or future. It posits that information can change from one form to another, but it cannot be created or destroyed, just like energy. That is, the total amount of information associated with the states and the changes of states of all physical entities in the whole universe at all times is fixed.

For the sake of discussion, let us assume that such a knowledgeable and capable *Laplacian intellect* existed at the time of the Big Bang, with full knowledge of the state of the baby universe at a certain instant, and with a full understanding of the laws and forces of physics. Since the envisaged Laplacian intellect possesses all the necessary means, such as memory, processing power, and post-processing capabilities, it would be able to analyze, predict, and mentally render the future accurately in full detail by tracing the actions and interactions of everything under the influences of the forces of physics in action within the bounds of the virtual template of the laws of physics.

That miraculous Laplacian mind would watch with amusement the entire universe unfolding and evolving before its eyes, from the formation of subatomic particles to the development of galaxies, stars, planets, and even the atmosphere in some planets as well as the formation of the winds and the rainfall. It would clearly see what the baby universe would grow into after billions of years. If the conservation of information is confined within the limits of the states of physical existence and the behavior of the physical entities under the influence of the laws and forces of physics as described above, there would be no points of contention since the physical information in this case would simply be the manifestation of the laws and forces of physics in the inanimate realm at the universal scale. 

However, when we consider the *living beings* and the animate realm at large, things get murky because of the enigmatic phenomenon of life, which is an anomaly since the emergence of life cannot be predicted by the laws of physics. That is, the wondrous Laplacian intellect with full knowledge of the laws and forces of physics that govern the inanimate realm would never be able to foresee the emergence of life. This is because life is not a natural outcome of the laws of physics, even under the most favorable conditions. If it could foresee it, we would now be creating life from nonlife right and left, like we are making new compounds and even elements, by manipulating inanimate existence and the laws of physics. 

Therefore, the emergence of life with its own causal power to subjugate matter in a purposive manner, and its taking center stage in existence, punches a major hole in Laplace’s deterministic thought, which works beautifully in the inanimate realm. As G. K. Chesterton [9] stated about a century ago “*A dead thing can go with the stream, but only a living thing can go against it. A dead dog can be lifted on the leaping water with all the swiftness of a living hound, but only a live dog can swim backwards*”.

No laws of physics can predict the next sentence that I will write, or what I will have for dinner. Additionally, no one turns to the laws of physics as the magical mirror globe to see what technological marvel will enter our lives next and how it will change the world. Instead, nations invest over 2 trillion dollars a year in research and innovation to create new information by empowering the most creative minds and hoping for an abundance of strokes of inspiration. For example, we are hopeful that we will have fusion reactors in the middle of this century, but it will not happen unless we first create the information on how to build such a reactor. 

In a world in which new information is the most valuable commodity and is protected by patents, copyrights, and trademarks, it is untenable to speak of conservation of information in a general sense. Therefore, the notion of conservation of information should be limited to the physical universe governed by the laws and forces of physics, and it should be referred to as *physical information* (or *orientational entropy*) to clearly distinguish it from other forms of information and knowledge. 

The physical information of a system involves both the *microscopic information* of the system at the constituent level, including the positions and velocities of particles within the bounds of the uncertainty principle, as well as the higher-level *macroscopic information* of the system, such as temperature and pressure at the system level. Microscopic information is related to the specification of the exact state of the constituents of a physical system, while macroscopic information characterizes the system as a whole. When a system is heated, for example, both the microscopic and macroscopic information will assume new values corresponding to the new state.

### 5.1. Limitations on the Conservation of Information 

The notion of conservation of information is often extended to include information that goes beyond physical information (or *orientational entropy*) encapsulated by the laws and forces of physics. For example, when a book is burned and the paper and ink are turned into smoke and ash that are dispersed, the information contained in the book is lost forever for all practical purposes. However, some zealous advocates of the conservation of information assert that the written information in the book at the macroscopic level is not really lost since, at least theoretically if not practically, the process can be reversed just like rewinding a video and playing it backwards, and the information can be recovered as the combustion products of the original book retrack the exact states during burning and recreate the original printed book.

However, the validity of this claim is not assured since it appears to violate the second law of thermodynamics: combustion is an irreversible process, and an irreversible process can never reverse itself. Doing so would result in the destruction of thermodynamic entropy, which is a violation of a fundamental law of physics. Therefore, the second law of thermodynamics renders the reverse process impossible. Of course, the process of the burning of the book supposedly can be forced-reversed by us by creating additional entropy externally and increasing the entropy of the universe. That is, barring technological difficulties, it is possible to artificially convert the smoke and ash particles of a book back into paper and ink. 

However, the only way we can reproduce the original book with the original typeset on its pages is to have a copy of the book in paper or digital form, or in the memory of a person. This is because irreversible processes do not have a unique process path that can be retracted. As a result, the best we can hope for by reversing the process is to recover the original material of the paper and ink from the smoke and ash using the principles of chemistry. Therefore, if the burned book was the only copy in existence with no storage in any form, including human memory as knowledge, then the information content of the book appears to be gone forever. Additionally, information in the ordinary sense is not doubled when a copy of a book is made—only the symbols of information that serve as the storage medium are doubled.

To underscore the point made above, now consider two physically identical books printed with identical amounts of ink and paper, except that one of them is a physics book while the other is on history. Now, these two books are burned in two different combustion chambers, producing an exact amount of smoke and ash with identical chemical compositions. If a chemist is called to duty to reconstruct the books, all the chemist can do is to retrack the chemical processes and recover the papers and the ink in each of the original books, which are identical. There is no way the chemist or anyone else can turn the first batch of paper and ink into a physics book and the other into a history book, and thus recover the original information. 

The smoke and ash have no signature of the ordering of the letters, words, sentences, and pages of the original book in the particular way they are organized, and thus they cannot be retracted. In fact, even the recovery of the paper and ink is not assured since the combustion of different materials may produce the same composition of smoke and ash. The recovery of information and thus the underpinning conservation of information does not seem tenable in this case since there is not a unique trajectory between the smoke-and-ash and the printed book, and thus the original printed book cannot be recreated. 

As another example, the thermal radiation that we all emit from our bodies is the outcome of our body temperature, which is partly the result of the food that we consume. However, no one can recover the information about what food I consumed today by simply examining the thermal radiation that my body is emitting. Additionally, we cannot predict the food and drinks a person has consumed by analyzing his or her breath since a variety of metabolized foods will result in the identical compositions of the breath, including the fractions of carbon dioxide and water vapor.

As a more intriguing example: current technological difficulties aside, how tenable is it to think that the ash and smoke produced by the incineration of the body of a person can be used as the constituents to rebuild the body in the exact way the person looked before incineration using only the signatures of information in the smoke and ash? To add spice to the question: What would the answer be if the person were alive beforehand?

### 5.2. Conservation of Information in the Animate Realm

We cannot speak of conservation of information associated with a system unless every state of the system uniquely specifies the state before and the state after in a deterministic way. That is, the possible states of a system must form a unique trajectory (a loop in the case of a cycle) that can be tracked back and forth as the configuration of the constituents of the system changes for the conservation of information to apply. Any deviation from the trajectory indicates information created or destroyed. That is, if we are moving with a system and information is conserved, we will always know exactly where we have come from and where we will go. In that case, the differences between states will propagate with time in a predictable manner as a consequence of the conservation of information. 

As a system evolves over time by undergoing a change from one configuration to another forward or backward, it remains on the same trajectory or cycle if information is conserved. Information is not conserved if a system remains on the same trajectory or cycle while going forward but not backward, and thus the system loses track of where it came from. Therefore, *reversibility* is a key feature for the conservation of information to apply. As such, the principle of conservation of information is limited to physical information under certain circumstances and should not be generalized over all information under all circumstances.

As an example, consider a healthy natural seed and an exact artificial replica of it such that both are chemically identical and indifferentiable. If we bury both seeds into moist soil, we know that the natural seed endowed with elusive life will germinate and sprout while its lifeless artificial twin will just sit there as a capsule of chemicals and disintegrate in time. Considering that these two seeds are physically identical and thus at the same state, their information (and entropy) content is the same. Additionally, the only difference between the natural and artificial seeds is the enigmatic life, which obviously is an elusive nonphysical existence. The changes that the artificial seed undergoes in a given environment can be predicted based on the universal laws of physics, but this is not the case for the natural seed. In general, all naturally occurring processes are irreversible, and thus they result in entropy (and thus information) generation. Only in the idealized case of all changes within the artificial seed occurring reversibly can we speak of the conservation of information (and thus entropy). 

In the case of the artificial seed, even the DNA molecule will be treated as an ordinary chemical, and it will have no influence on the changes occurring within the seed. In the case of the natural seed, however, the same passive DNA molecule, which is a set of instructions inscribed by the genetic letters of A, C, G, and T, turns into an active player under the apparent action of elusive life, and serves as a template or strict code of conduct for all occurrences within the domain of life. This is like a passive recipe acting as an invisible chef when it acquires life. A tomato seed, for example, will sprout into a tomato plant with well-defined boundaries in a purposive way, rather than undergoing random changes under the laws of physics alone, like the artificial seed.

It appears that when a healthy natural seed is planted into soil, there *is maximum certainty* about what will happen to the seed and the materials in its vicinity, and thus *minimum information* and *entropy*, since certainty is inversely proportional to entropy. As live entities, seeds germinate and sprout when planted, and thus undergo internal changes that cannot be foreseen by the universal laws of physics. The changes that a plant seed undergoes during germination are specific to its species, and denying life because we cannot observe it and do not understand it is equivalent to denying all the differences between a healthy natural seed and its physically identical artificial twin. We cannot have an accurate and comprehensive understanding of information in the physical realm unless the supremacy of the enigmatic life in the animate realm is properly considered.

Similarly, a fertilized chicken egg placed in an egg incubator maintained at a temperature of 38 °C will hatch in about 21 days, and the white and yolk in the egg will turn into a live chick complete with eyes, legs, wings, and colorful fur. If, on the other hand, the egg is left unattended, we know that the white and yolk of the egg will disintegrate and turn into a near homogeneous liquid mixture with the familiar rotten-egg smell. 

Consider two identical eggs, each with trillions of atoms and molecules, at the same state or configuration. One egg is placed in an egg incubator while the other is left unattended in an environment with varying conditions. Initially, both eggs have the same amount of information or entropy since they are physically identical and exist at the same state. The universe of the possible configurations *N* of the atoms and molecules within either egg at any instant constitutes the total information *I* or entropy *S* for that egg. However, the information contents of the two eggs will deviate considerably as a chick is built in one egg while random purposeless chemical reactions take place in the other. Therefore, when it comes to information or entropy in the physical realm, which is comprised of animate and inanimate realms, the presence or absence of life is the dominant factor. This point of view may also shed light on the debate on whether a virus is a living being or not.

When the possible chemical reactions are considered, the total number of configurations *N* is extremely large, and thus the amount of information or entropy is very large—but finite. In the case of the egg in the incubator, the probability of the contents of the egg assuming the exact configuration of the chick is very high (close to 1), while all other probabilities are near zero. Therefore, we can say that there is *maximum certainty* that the egg in the incubator will turn into a live chick, and thus minimum entropy and information associated with the final state of the egg within the incubator. However, there is large uncertainty about the distribution of microscopic states within the rotten egg, and a large amount of entropy and information associated with it. 

A more fundamental problem associated with information is the confusion of information with the symbols of information—the physical media that serve as the carrier and represent the information using a common code like a language that matches symbols with meanings. For example, a typical paragraph on this page has 500 letters, and it is perceived as information by a person who reads it since the letters are organized in an intelligible way and thus it is meaningful. However, if all the English-speaking people in the world were suddenly to disappear, that same paragraph would no longer contain any information—it would be perceived as an array of letters in a certain pattern. If we take those 500 letters and randomly align them in groups of 1 to 10 letters, the new arrangement will look just like a paragraph from a distance. However, when closely examined, it will be labeled as garbage instead of meaningful information. 

Therefore, the patterns of physical symbols, molecules, sounds, or any other entities are not meaningful information unless there is meaning associated with that arrangement. The air molecules in the room move continually and exhibit new patterns as they rearrange, but the only information they exhibit is the physical information related to the positions, speeds, collusions, etc. of the air molecules themselves that completely specify the state of the air in the room.

In summary, the notion of conservation of information is limited to the domain of the microscopic structure of physical beings, and the *physical information* related to the states and behaviors of the constituents of the physical entities. In the subatomic world, the physical information is referred to as *quantum information,* which is associated with the quantum states of particles, such as the wave function, and calculated using the probabilistic definition of entropy, as in communication theory. Yet, new information emerges in quantum mechanics within the context of entanglement.

## 6. Closure

Information in the communication field is of a statistical nature, and it represents probabilities. Information associated with a message is intimately related to the probability of that message. Information is a measure of the choices we have for a message. It is related to the number of possible different messages or configurations associated with a system, not how much we know about the system. Information or entropy is related to the probability distribution associated with the internal structure or the configuration of the constituents of a system and represents the degree of incompleteness of our knowledge about the precise state of the system under consideration. In a counterintuitive sense, the more we know about a system, the less remains to be known about that system, the less the uncertainty, the fewer the choices, and thus the smaller the information (and entropy). 

Information reaches its maximum value when the probabilities of all states are equal. This corresponds to the case of maximum information, maximum uncertainty, maximum entropy, and minimum knowledge. That is, when there are many choices, information is the largest when the probabilities of choices are nearly uniformly distributed, making every choice almost equally viable. Information is smallest when one of the choices has a probability of nearly one, rendering other choices nil and thus leaving little freedom of choice. 

The formulations of statistical thermodynamic entropy and informational entropy differ only by a constant factor. Due to the close resemblance of their mathematical definitions, entropy and information are often used interchangeably. However, they have different units, and thus they represent different quantities. 

Energy, entropy, and information are all additive quantities. However, energy is always conserved during a process, informational entropy is conserved only during transmission when there are no interventions from noise or other causes, and thermodynamic entropy is almost always generated during a process except for the rare cases of reversible processes such as superconductivity. 

The additive nature of entropy at a given state should not be confused with the conservation of entropy since the concept of conservation is associated with a change of state or a process, not a particular state. When an isolated system undergoes a change, the total energy content of the system remains constant while the entropy increases as a result of the irreversibilities that occur during the process. This is why we have the *conservation of energy principle* and the *increase of entropy principle* in thermodynamics. 

The notion of *conservation of information* with the implication that there is a fixed amount of information in the universe is not in line with our intuitive understanding of information since we are overwhelmed by the amount of new information, and the continual acquisition of knowledge. The near-synonymous use of knowledge and information in daily life and the intuitive sense that knowledge is limitless adds to the confusion associated with this notion. Therefore, it is important to set clear boundaries, and put different definitions of information into a proper perspective.

The laws and forces of physics serve as the template for the physical world, making the behavior of physical entities under external influences predictable. Therefore, as Laplace stated a few centuries ago, the overarching information behind the changes of state in the physical universe is the knowledge of the laws and forces of physics, which are unchanging. Once we have accumulated all the knowledge associated with the laws and forces of physics, we cannot gather any new information that cannot be predicted by the complete set of the laws of physics by simply examining the intense activity and changes in the physical world. Conservation of information is tenable only in this limited sense. Then, all the physical activity becomes a beautiful display or play-out of these fundamental laws and forces, and we can say that the information about the state of the physical universe is conserved over time.

## Data Availability

The study did not report any data.
